# On Floodings

**Published:** 1846-10

**Authors:** 


5. *On Floodings.*—"1. Floodings rarely occur after natural delivery, to any extent, if properly guarded against. 2. They happen most frequently after instrumental and manual deliveries, and after deliveries rendered precipitate by the violence of the expulsive action, *in all of which cases they proceed from lacerations of the soft parts*, sustained during the passage of the child. 3. Those which occur after labors rendered tedious by the abnormal size of the child, may proceed either from laceration or sloughing of the parts. Some rare cases are on record, in which the blood would seem to have escaped by gravity from the uterine vessels, owing to the mother having been raised into an erect posture while in a debilitated state.* 4. Floodings which take place a few hours after delivery, are owing to wounded vessels which have acquired increased activity after the depression occasioned by the shock of delivery has gone off. 5. Those which take place some days after delivery, are connected with sloughing of the parts, which may either have been injured in the act of delivery, or become tainted by the presence of a putrid portion of the placenta." The phenomena of floodings being thus shown to be identical with those of hemorrhages from wounded arteries, the same plan of treatment is clearly identical in both cases. Floodings

* How can this be, unless there are uterine vessels communicating with the placenta?

then, are to be treated by exposure to cool air, by cold applications to the parts, or, if need be, by cooling injections into the uterus and vagina, by elevated position of the pelvis, and moderate doses of opium. If arterial blood flow rapidly and continuously, an examination should be made, and if a wounded artery is detected, it should be secured by the usual surgical means.*—*London Medical Gazette*, in *New York Journal of Medicine.*

* Medical Gazette, vol. xxxvii., p. 150.

